# Integrated trauma care successfully treats a severe case of combined steel bar penetration injury to the head, neck, chest, and abdomen: A case report and literature review

**DOI:** 10.1097/MD.0000000000043529

**Published:** 2025-08-08

**Authors:** Jiangling Yao, Xinyue Zheng, Jian Yang, Yuxi Huang, Xingchen Ming, WeiHao Xiao, Shaowen Cheng, Yunfu Zeng, Hong Huang, Yangyang Bian

**Affiliations:** a Key Laboratory of Emergency and Trauma of Ministry of Education, Wound Repair Department, Key Laboratory of Hainan Trauma and Disaster Rescue, The First Affiliated Hospital, Hainan Medical University, Haikou, China; b Hainan Medical University, Haikou, China.

**Keywords:** damage control surgery, multidisciplinary care, penetrating trauma, polytrauma, trauma resuscitation unit

## Abstract

**Rationale:**

Penetrating injuries spanning multiple anatomical regions carry significant risks of morbidity and mortality. Successful management depends on coordinated prehospital care, expedited imaging, and a multidisciplinary surgical approach. This case report details the successful management of an exceptionally severe combined penetrating injury involving the head, neck, chest, and abdomen, emphasizing the critical role of an integrated trauma care system. It serves to highlight the importance of standardized trauma protocols for optimizing outcomes in such complex and life-threatening injuries.

**Patients concern and diagnosis:**

The patient exhibited acute distress, hemodynamic instability, and 2 penetrating steel rebars traversing the head, neck, chest, and abdomen. Computed tomography (CT) scans revealed trajectories through critical structures, including the right orbit, cervical vascular sheath, and intra-abdominal organs, prompting immediate surgical intervention.

**Interventions:**

Emergency response: the trauma resuscitation team was activated, followed by fluid resuscitation and urgent CT imaging. Surgical procedures: (1) Open wound debridement and foreign body removal; (2) diaphragmatic repair and liver laceration management; (3) closed thoracic drainage and clavicle fracture fixation; (4) postoperative care: intensive monitoring, infection prevention, and rehabilitation.

**Outcomes:**

(1) Successful stabilization and foreign body extraction; (2) full recovery with near-complete functional restoration by postoperative day 20; (3) no significant complications observed during follow-up.

**Lessons:**

(1) Multidisciplinary collaboration and standardized trauma protocols are vital for complex cases; (2) CT imaging remains indispensable despite metallic artifacts; (3) institutional preparedness and continuous team training improve trauma care efficacy.

**Key points**

• Rapid activation of a multidisciplinary trauma team is critical for managing complex penetrating injuries.
• Preoperative imaging, particularly CT scans, plays a vital role in surgical planning despite artifact challenges.
• Integrated trauma care systems significantly improve survival and reduce complications in polytrauma patients.
• Continuous training and standardized protocols enhance trauma team efficiency and patient outcomes.

## 1. Introduction

Penetrating trauma to the head, neck, chest, abdomen, and scrotum represents a complex and severe form of injury that poses significant challenges in emergency medicine. These injuries typically arise from high-energy mechanisms like falls from heights or motor vehicle collisions, frequently causing multisystem organ damage and major vascular disruption. Patients often present with hemorrhagic shock, neurological impairment, or hemodynamic instability, demanding rapid multidisciplinary intervention.^[1,2]^ The management of such cases requires a well-structured trauma care system that integrates prehospital emergency services, surgical intervention, and postoperative care to optimize patient outcomes and minimize morbidity and mortality associated with these life-threatening conditions.^[3]^

This case report details the management of a 19-year-old male who sustained a rare and severe combined penetrating injury after falling from a height of approximately 6 meters, resulting in 2 steel rebars penetrating through his head, neck, chest, and abdomen. This case is particularly significant as it highlights the critical need for a comprehensive trauma response system capable of addressing the complexities associated with such multifaceted injuries. The report serves as an important reminder for clinicians to consider similar high-energy penetrating injuries in patients presenting with acute trauma, especially in construction or industrial settings where such accidents are prevalent.

## 2. Case presentation

On October 5, at 06:30, emergency surgical physicians at the First Affiliated Hospital of Hainan Medical University received an emergency alert regarding a patient with penetrating trauma to the head, neck, chest, and abdomen due to steel rebar. A 19-year-old male worker fell from a height of approximately 6 meters at a construction site, resulting in 2 steel rebars penetrating his body: one rebar entered through the left back, traversed the left mandible, and exited through the right eye socket, while the other entered the left anterior chest and exited through the left posterior back. Ethical approval for this case report was granted by the Ethics Committee of the First Affiliated Hospital of Hainan Medical University. Written informed consent was obtained from the patient and his family.

### 2.1. Emergency response

Upon receiving the alert, the emergency department and trauma medical center staff quickly assembled and activated the trauma resuscitation unit, designed for severe trauma cases, equipped with life support devices such as ventilators, vital parameter monitors, and defibrillators. The medical department coordinated with various specialties, including trauma intensive care unit (TICU), neurosurgery, otolaryngology, operating room, anesthesia, oral surgery, and ophthalmology, while also liaising with the fire department for preparedness. A robust trauma resuscitation team was ready for immediate intervention.

**Figure 1.**
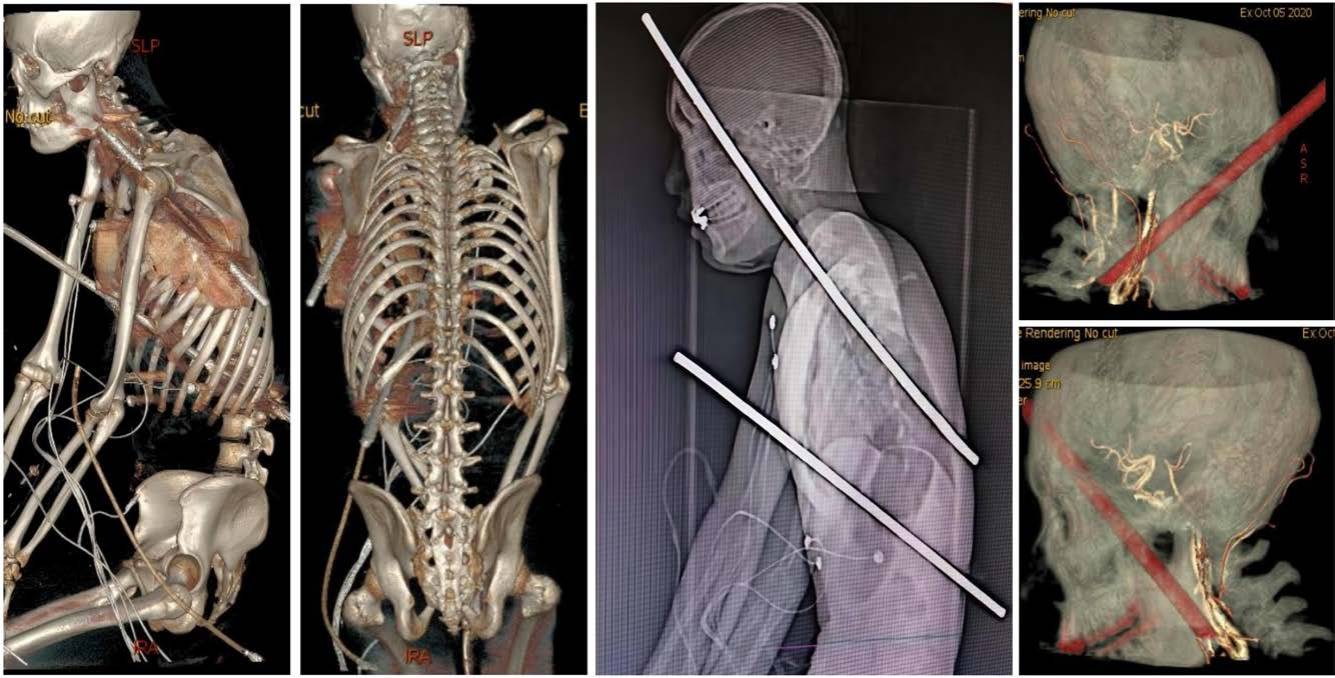
The trajectory of the steel rebar, as confirmed by CT imaging, traversed from the right frontal region, lateral to the right eye socket, into the temporal fossa, through the pterygoid fossa and sphenoid wing, into the oropharynx, along the right cervical vascular sheath, into the left supraclavicular fossa, and into the left axilla. The rebar also penetrated the left fifth costal cartilage, traversing along the greater curvature of the stomach, the splenorenal space, and exiting below the left twelfth rib at the lateral posterior abdominal wall. CT = computed tomography.

The patient was brought to our hospital by Haikou City 120 Emergency Center at 06:57, presenting with severe distress, restlessness, and early signs of shock. The attending physician promptly opened the trauma green channel for life support monitoring and treatment, conducted rapid injury assessment and bedside examinations, initiated fluid resuscitation, and prepared for surgery. At 07:02, a comprehensive full-body computed tomography (CT) scan was performed to evaluate the condition, and the patient was swiftly transported to the operating room. The trajectory of the steel rebar, as confirmed by CT imaging, traversed from the right frontal region, lateral to the right eye socket, into the temporal fossa, through the pterygoid fossa and sphenoid wing, into the oropharynx, along the right cervical vascular sheath, into the left supraclavicular fossa, and into the left axilla. The rebar also penetrated the left fifth costal cartilage, traversing along the greater curvature of the stomach, the splenorenal space, and exiting below the left twelfth rib at the lateral posterior abdominal wall (Fig. 1).

### 2.2. Diagnosis

Multiple injuries (penetrating wounds of head, neck, chest, and abdomen).

1. Multiple injuries (penetrating wounds of head, neck, chest, and abdomen)
  1.1. Penetrating head and neck injury with retained rebar foreign body
  1.2. Penetrating chest and abdomen injury with retained rebar foreign body
  1.3. Rupture of left diaphragm
  1.4. Laceration of left hepatic lobe
  1.5. Slight pneumothorax in left thoracic cavity
  1.6. Fracture of middle segment of left clavicle
  1.7. Fracture of lateral and posterior walls of right maxillary sinus, and fracture of right pterygoid process
  1.8. Injury to left brachial plexus

### 2.3. Surgical intervention

At 07:22, the trauma medical center’s esteemed expert led the surgical team in collaboration with Professor Yang from the neurosurgery department, alongside representatives from oral surgery, otolaryngology, ophthalmology, and the anesthesia team. Based on the patient’s injuries, a damage control surgery was executed, which included:

- Open wound debridement and suturing of head and neck penetrating injuries
- Removal of steel foreign bodies from the head, neck, chest, and abdomen
- Exploration of the left chest and abdomen for penetrating injuries
- Repair of the left hemidiaphragm
- Repair of a 2.0 cm × 2.0 cm laceration of the left lobe of the liver
- Closed drainage of the left thoracic cavity

Exploration revealed no significant damage to the intestines, spleen, stomach, or left lung. However, a 2.0 cm × 2.0 cm contusion of the left lobe of the liver and a 2.0 cm rupture of the left hemidiaphragm with minor hemorrhage and contamination were noted.

### 2.4. Postoperative care

**Figure 2.**
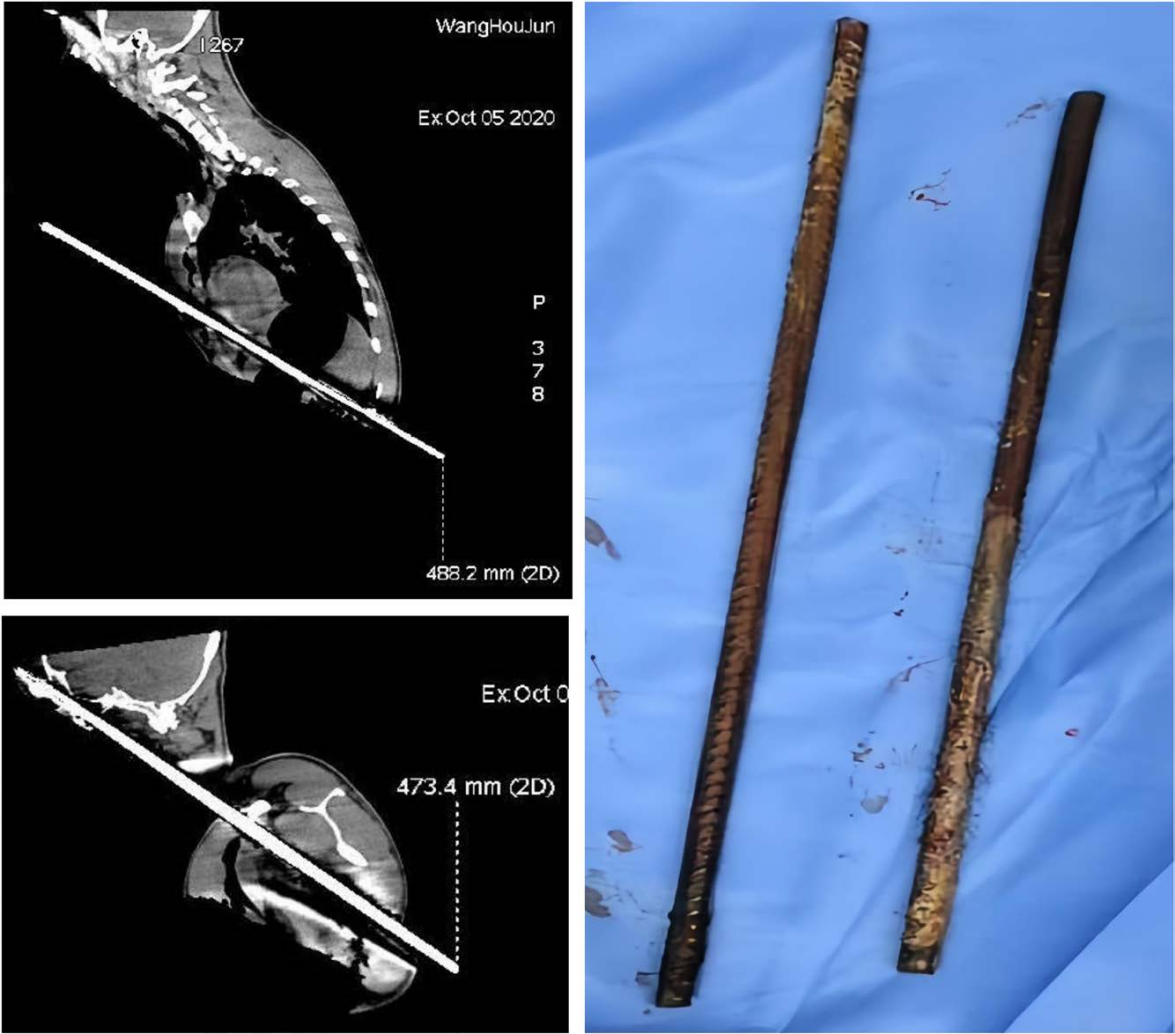
The surgery was successful in removing 2 steel bars, measuring approximately 488.2 mm and 473.4 mm in length.

At 12:55, the surgical procedure was successfully completed. Two steel bars with approximate lengths of 488.2 mm and 473.4 mm were extracted intraoperatively (Fig. 2). At 13:00, the patient was transferred to the TICU postoperatively under sedative-analgesia, with endotracheal intubation and invasive mechanical ventilation in Assist/Control (A/C) mode, where the patient was subjected to further life-support therapy and prophylactic measures against postoperative complications. On October 9, after confirmation of stable vital signs, the patient was transferred back to the Trauma Medical Center for continued postoperative management. On October 23, Professor Peng performed open reduction and internal fixation for the patient’s left clavicular fracture.

## 3. Follow-up and outcomes

**Figure 3.**
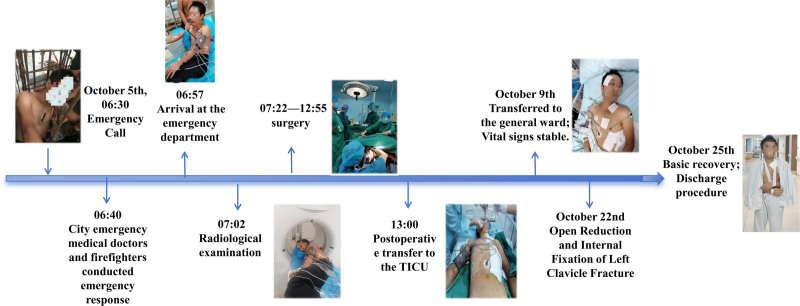
The whole treatment process of patients.

On October 25, the patient demonstrated significant recovery. The penetrating wounds exhibited satisfactory healing without signs of infection. Neurological assessment revealed near-complete resolution of deficits except for residual brachial plexus injury in the left upper limb. This manifested as sensory impairment (reduced light touch and pinprick sensation) in the medial forearm, ulnar aspect of the palm, and ulnar dorsum of the hand, accompanied by weakness in shoulder flexion and external rotation (Medical Research Council grade 4/5). Motor function in the right upper limb and bilateral lower limbs was intact. The patient was mobilized effectively and achieved independence in activities of daily living. The entire treatment process is illustrated in Figure 3. The patient was discharged on the 20th day post-admission. After 3 months of subsequent outpatient monitoring, no significant complications, such as surgical site infection, vascular complications, or delayed neurological deterioration, were reported. The brachial plexus injury showed gradual improvement with ongoing physiotherapy.

## 4. Discussion

The rarity of combined penetrating trauma makes this case clinically significant, while also illustrating the complex challenges posed by such injuries. The 19-year-old male patient, sustaining injuries from a rebar penetrating multiple anatomical regions, exemplifies the critical nature of trauma that can arise from industrial accidents.^[4]^ This case is particularly noteworthy as it highlights the need for an integrated trauma response system capable of addressing the complexities and rapid progression of such injuries. The literature indicates that penetrating injuries involving the head, neck, chest, and abdomen often lead to significant morbidity and mortality if not managed promptly and effectively.^[5,6]^ The trauma medical center’s rescue model is distinct from conventional methods, providing an integrated and systematic approach to patient care.^[7]^ Upon receiving the emergency call, prehospital physicians accompany the ambulance to the site for immediate on-scene interventions, such as resuscitation and fluid administration. In cases of critical conditions, they communicate with in-hospital emergency physicians to prepare for the patient’s arrival.

Preoperative imaging plays a critical role in steel bar penetrating injuries, where the wound tract may extend deeply with unpredictable tissue damage.^[8]^ While CT serves as the primary modality for detecting retained foreign bodies, hematomas, and osseous fragments, beam-hardening artifacts from high-density metallic objects often limit diagnostic accuracy, sometimes requiring supplemental imaging studies.^[9]^ In this instance, timely CT results indicated that the metallic foreign body was closely associated with the left common carotid artery bifurcation, with indistinct boundaries due to artifact interference, guiding subsequent surgical planning.

Current literature on penetrating trauma demonstrates that effective management demands multidisciplinary collaboration. A robust emergency rescue system (integrating prehospital care, trauma resuscitation units, operating rooms, TICUs, and general wards) provides comprehensive treatment for severe injuries.^[10]^ Thus, it is crucial to have a well-established integrated trauma rescue system, standardized trauma management processes, and an efficient trauma care team capable of delivering timely and effective emergency interventions to preserve life and reduce mortality and morbidity rates. Existing guidelines recommend a coordinated effort among trauma teams to ensure that interventions are timely and comprehensive.^[11]^ The rapid assessment and subsequent surgical intervention in this case reflect current best practices in trauma management, where the establishment of trauma systems and protocols can significantly impact survival rates.^[12,13]^

This report demonstrates that structured multidisciplinary collaboration significantly improves trauma care efficacy. The coordinated efforts across departments at Hainan Medical University First Affiliated Hospital provide a replicable model for similar institutions. Continuous team training and simulation remain critical for optimizing performance during high-acuity scenarios, as evidenced by enhanced team dynamics and patient outcomes.^[14]^ Furthermore, this case emphasizes the necessity for trauma centers to maintain a high level of preparedness for complex cases, including the availability of essential resources and a robust trauma care protocol.^[15,16]^ The establishment of trauma response units, as noted in the current case, is a vital component of modern trauma systems that can facilitate the rapid delivery of care, ultimately improving patient outcomes.^[17]^

In conclusion, this case underscores the need for sustained education and training in trauma care within integrated response systems. Strengthening interdisciplinary collaboration enhances both immediate patient management and long-term advancements in trauma care delivery. These findings support further development of adaptable, standardized trauma protocols to reduce mortality and improve care quality across diverse healthcare settings.^[18]^

### 4.1. Limitations

This report details a single, albeit successful, case. As a case report, it inherently lacks comparative data or statistical power to generalize findings. The conclusions drawn regarding the efficacy of the integrated trauma system are observational and specific to this unique clinical scenario. Furthermore, while CT imaging was crucial, beam-hardening artifacts from the steel rebars limited the precise delineation of the foreign body’s relationship to adjacent critical neurovascular structures preoperatively.

## Acknowledgments

We acknowledge DeepSeek for its assistance in translating and refining the manuscript and the anonymous reviewers for their constructive comments.

## Author contributions

**Conceptualization:** Jiangling Yao, Shaowen Cheng, Yunfu Zeng, Yangyang Bian.

**Funding acquisition:** Jiangling Yao, Yuxi Huang, Shaowen Cheng, Hong Huang, Yangyang Bian.

**Investigation:** Jiangling Yao, Jian Yang, WeiHao Xiao.

**Methodology:** Jiangling Yao, Yuxi Huang.

**Resources:** Jian Yang.

**Writing – original draft:** Jiangling Yao, Xinyue Zheng, Xingchen Ming, WeiHao Xiao, Hong Huang, Yangyang Bian.

**Writing – review & editing:** Shaowen Cheng, Yunfu Zeng, Hong Huang, Yangyang Bian.
